# Exploring the aging process of cognitively healthy adults by analyzing cerebrospinal fluid metabolomics using liquid chromatography-tandem mass spectrometry

**DOI:** 10.1186/s12877-023-03939-6

**Published:** 2023-04-05

**Authors:** Fu-Chao Liu, Mei-Ling Cheng, Chi-Jen Lo, Wen-Chuin Hsu, Gigin Lin, Huan-Tang Lin

**Affiliations:** 1 Department of Anesthesiology, Chang Gung Memorial Hospital, 5 Fu-Shin Street, Kwei-Shan Taoyuan, 333 Taiwan; 2 College of Medicine, Chang Gung University, Taoyuan, 333 Taiwan; 3 Metabolomics Core Laboratory, Healthy Aging Research Center, Chang Gung University, Taoyuan, 333 Taiwan; 4 Department of Biomedical Sciences, College of Medicine, Chang Gung University, Taoyuan, 333 Taiwan; 5 Clinical Metabolomics Core Laboratory, Chang Gung Memorial Hospital, Taoyuan, Taiwan; 6 Department of Neurology, Chang Gung Memorial Hospital, Taoyuan, 333 Taiwan; 7 Department of Medical Imaging and Intervention, Imaging Core Lab, Chang Gung Memorial Hospital, Taoyuan, 333 Taiwan; 8 Graduate Institute of Clinical Medical Sciences, College of Medicine, Chang Gung University, Taoyuan, 333 Taiwan

**Keywords:** Aging process, Cerebrospinal fluid, Metabolomics, Liquid chromatography-mass spectrometry

## Abstract

**Background:**

During biological aging, significant metabolic dysregulation in the central nervous system may lead to cognitive decline and neurodegeneration. However, the metabolomics of the aging process in cerebrospinal fluid (CSF) has not been thoroughly explored.

**Methods:**

In this cohort study of CSF metabolomics using liquid chromatography-mass spectrometry (LC–MS), fasting CSF samples collected from 92 cognitively unimpaired adults aged 20–87 years without obesity or diabetes were analyzed.

**Results:**

We identified 37 metabolites in these CSF samples with significant positive correlations with aging, including cysteine, pantothenic acid, 5-hydroxyindoleacetic acid (5-HIAA), aspartic acid, and glutamate; and two metabolites with negative correlations, asparagine and glycerophosphocholine. The combined alterations of asparagine, cysteine, glycerophosphocholine, pantothenic acid, sucrose, and 5-HIAA showed a superior correlation with aging (AUC = 0.982). These age-correlated changes in CSF metabolites might reflect blood–brain barrier breakdown, neuroinflammation, and mitochondrial dysfunction in the aging brain. We also found sex differences in CSF metabolites with higher levels of taurine and 5-HIAA in women using propensity-matched comparison.

**Conclusions:**

Our LC–MS metabolomics of the aging process in a Taiwanese population revealed several significantly altered CSF metabolites during aging and between the sexes. These metabolic alterations in CSF might provide clues for healthy brain aging and deserve further exploration.

**Supplementary Information:**

The online version contains supplementary material available at 10.1186/s12877-023-03939-6.

## Introduction

Over the past 60 years, improvements in healthcare and a booming economy have led a marked increase in the average human lifespan by almost 23 years [1]. According to the latest *World Population Ageing Report* published by United Nations, the size of the population aged above 65 will increase by 120% from 0.7 billion in 2019 to 1.5 billion in 2050, and 64% of the aged population in 2050 will be living in Asia [1]. Progressive metabolic dysregulation is a generalized hallmark of biological aging. Because the brain metabolizes approximately one-quarter of systemic glucose for energy production but constitutes only 2% of body weight, these metabolic changes may have an exaggerated presentation in the brain [2]. With aging, there is an exponential risk of cognitive decline and neurodegenerative diseases. Common neurodegenerative diseases caused by accumulated misfolded protein aggregates, such as Alzheimer’s disease (AD) and Parkinson’s disease (PD), have become a major challenge for the next generation, and unfortunately, there is almost no curative treatment to date. Therefore, obtaining deeper insights into the healthy aging process in the brain and exploring novel strategies to achieve healthy brain aging and prevent neurodegenerative diseases are urgently needed [3].

During brain aging, dysregulated bioenergetics, neuroplasticity, and neuroinflammation contribute to the risk of cognitive decline and neurodegenerative disorders [4]. Age-related accumulation of oxidative stress may lead to functional decreases in cerebral energy metabolism, including glucose transport, mitochondrial oxidative phosphorylation, DNA repair, and redox regulation [4]. Brain hypoperfusion and blood–brain barrier (BBB) leakage in elderly individuals can contribute to diminished nutrient import and toxin removal, leading to cognitive decline [5–7].

Aging-related degradation of molecular and cellular processes contribute to genome, proteome, and lipidome instability, and the minor changes of these upstream molecules can cause significant metabolite alterations [8]. Because perturbations in metabolic pathways can be one of the first measurable alteration before disease manifestations, metabolomics can be used to characterize the dynamic biological aging processes. Previous aging-related metabolomic studies of various biofluids, such as blood samples (serum or plasma), urine, and saliva, obtained from model organisms and humans demonstrated that aging-related metabolites are mostly associated with carbohydrates, lipids, amino acids, DNA repair, and redox metabolism [9–11]. For example, a plasma-based metabolomics analysis of the aging process showed that ceramide, fatty acids, methionine, and nitric oxide pathways are associated with healthspan in healthy adults [12]. Age-specific metabolic fingerprints differ significantly by sex, with a substantial atherogenic transition overlapping menopause in females [13]. A large-scale (26,050 adults) Northern European study on the effects of age, sex, and menopause using serum metabolomics found that menopause status is associated with significant amino acid and lipid alterations, which might contribute to future metabolic and cardiovascular risks in females [13]. Different rates of cognitive decline and brain atrophy between men and women have been observed in patients with AD, but whether that sex differences in brain aging trajectories exist during the process of healthy human brain aging requires further exploration [13, 14].

Cerebrospinal fluid (CSF) is an appropriate biospecimen for analyzing the aging of the central nervous system (CNS), because CSF interacts closely with CNS tissue and its composition can reflect brain-specific metabolite alterations during aging. CSF exchanges metabolites between the cerebral and systemic circulation; however, the specialized tight junctions of the BBB limit transcellular transport in the CNS [15]. Although CSF has been profiled in the context of various neurological diseases to provide novel insights into disease mechanisms, the CSF metabolomics of the healthy aging process has not been thoroughly explored [16, 17]. The earliest CSF metabolomics analysis of normal individuals was conducted in 2010 in a small study of 10 adults that compared CSF proteomics and metabolomics and found high metabolomic variation among individuals [18]. A recent small Swedish study using liquid chromatography mass spectrometry (LC–MS) to explore CSF metabolomics during healthy aging found several aging-related metabolites, but further larger cohort studies are required because of the small number of cases (23 individuals) and limited patient information (claimed healthy but no medical examination) [19]. Our previous nuclear magnetic resonance (NMR) metabolomic study showed that the CSF alterations in citrate, lactate, leucine, tyrosine, and valine had a good correlation with the aging process [20]. To further quantify the delicate metabolic alterations during healthy brain aging, we conducted this LC–MS metabolomic study in a larger population of cognitively healthy patients to profile the metabolic alterations in CSF during aging and between sexes.

## Materials and methods

In the current metabolomic study, the LC–MS metabolomic profiles of CSF samples collected from cognitively healthy patients were analyzed to examine the metabolomic alterations during the aging process and between different sex. This clinical study was deposited in the Clinical Trials Registry (ClinicalTrials.gov Identifier: NCT04315038, first registered on 19/03/2020) and approved by the Institutional Ethical Review Board (approval number: 201801931A3). We have explicitly explained the study protocol to every participant before enrollment into the study, and written informed consent was obtained after explanation.

### Study population

We enrolled adult participants who were cognitively healthy (without neurological or psychiatric diseases) and were receiving optional spinal anesthesia for elective surgery at Linkou Chang Gung Memorial Hospital, a tertiary medical center in Northern Taiwan. The study participants were divided into three groups by age: young (age 20–39 years), middle-aged (40–59 years), and old (aged ≥ 60 years). The three-age group classification has been applied in previous LC–MS metabolomic study of human aging [21]. Patients with a history of diabetes and obesity were excluded from the final analysis because insulin resistance in these patients could confound the metabolite alterations [22]. From June 1, 2019 to March 31, 2020, a total of 100 participants completed the initial screening and underwent CSF sampling for metabolomic analysis. All participants were admitted for elective urological or orthopedic surgeries and fasted for ≥ 8 h before CSF sampling.

Cognitive evaluation was based on preoperative assessment and the exclusion of neurological or psychiatric diseases; thus, mild cognitive impairment might have been overlooked. After the initial evaluation, we excluded eight participants with a body mass index (BMI) > 30 kg/m^2^ or a fasting blood glucose > 126 mg/dL from the analysis because these patients were considered obese or diabetic, respectively, according to diagnostic criteria [23]. The final cohort consisted of 34, 31, and 27 patients in the young, middle-aged, and old age groups, respectively. Demographic characteristics, including age, sex, body height, body weight, and laboratory examination results, were recorded and compared. Other biochemical data, such as plasma glucose and serum creatinine levels, were recorded from the laboratory results before CSF sampling.

### Collection of CSF samples

The CSF collection procedures were largely the same as that described in our previous NMR metabolomic study [20]. After obtaining informed consent, we collected CSF samples during the routine spinal anesthesia procedure using a 26-gauge spinal needle at the L3–L4 or L4–L5 interspace. After free flow of clear CSF from the spinal needle, 1.2 mL of CSF was drained into a polypropylene tube, aliquoted, and stored at -80 °C until analysis. No immediate complications or patient discomfort were reported during CSF sample collection.

### Sample preparation and non-targeted LC–MS metabolomics

The CSF samples (50 μL) were mixed with cooled methanol (200 μL) to precipitate proteins. After centrifugation at 12,000 × g for 15 min, the supernatant was transferred to nitrogen gas for drying. The residue was suspended in 200 μL of 50% acetonitrile for LC–MS analysis.

Liquid chromatographic separation was conducted on an ACQUITY UPLC BEH Amide column (1.7 μm, 2.1 × 150 mm; Waters, Milford, MA, USA) using an ACQUITY TM Ultra Performance Liquid Chromatography (UPLC) system (Waters Corp.). The column was maintained at 45 °C, and the flow rate was 0.4 mL/min. The mobile phase consisted of 0.1% formic acid in water (phase A) and acetonitrile containing 0.1% formic acid (phase B). Mass spectrometry was performed on a Waters Q Tof–MS (SYNAPT G2S; Waters MS Technologies, Manchester, UK) operated in ESI positive and negative ion modes. The scan range was 50–1000 m/z. The desolvation gas flow rate was 800 L/hr at 500 °C. The source-cone voltage was set to 25 V. The capillary voltage was 2.5 kV in positive mode and 2 kV in negative mode. The lock mass was leucine encephalin (m/z: 120.0813 and 556.2771 for positive mode and m/z: 236.1035 and 554.2615 for negative mode).

### Statistical analysis

Finally, 162 CSF compounds were identified by LC–MS using an in-house library of CSF samples. The in-house library of CSF samples was set up by standard metabolite annotation or MS/MS fragment verification. Metabolite annotation was performed using accurate mass, retention time, and MS/MS criteria. Raw data can be obtained from the corresponding author. Of the 162 compounds, 37 CSF metabolites could be characterized using the CSF metabolome database of the Human Metabolome Database (HMDB) with high confidence [24]. Further multivariate analysis such as orthogonal projections to latent structures-discriminant analysis (OPLS-DA) model was performed using SIMCA-P + software (version 13.0; Umetrics, Umea, Sweden) under Pareto scaling. We utilized the variable Y of each metabolite from the constructed OPLS-DA model as the LC–MS signal integration to compare their metabolite abundance and calculate the Akaike information criterion (AIC) and area under the curve (AUC) for further fitting comparison. We then applied MetaboAnalyst 5.0, an online analytic tool, for metabolomic analyses, including heatmaps, enrichment analysis, and pathway analysis [25].

The recorded data are expressed as means ± SD for continuous variables and percentages for qualitative variables (sex and diseases). The statistical analyses in our study were based on the acquired LC–MS signal integration of each metabolite, and data were compared using Student’s t-test or χ^2^ tests (for two-groups) and analysis of variance (ANOVA) (for multiple groups). The between-group differences of a specific metabolite were compared using the OPLS-DA coefficients of the LC–MS signals, and the variables in the OPLS-DA score plots were compared using goodness of fit (R^2^X, R^2^Y, and Q^2^).

In this study, CSF metabolite profiling of cognitively healthy adults of different age groups was the primary outcome. The aim of this analysis was to identify CSF metabolites that could discriminate between the young and old age groups, and then calculate the correlation between metabolite abundance and aging using a regression model. Metabolites with significant discrimination between the old and young groups were selected to construct metabolite combinations; those combinations with lower AIC values, higher AUC values, or higher odds ratios (ORs) were selected to evaluate their discrimination of these two age groups. Between-group comparisons were adjusted for sex, BMI, serum creatinine level, and medical history of hypertension since these variables had significant between-group differences in the demographic comparisons. As the aging process might differ between the sexes, we calculated the correlation of each metabolite with aging in male and female adults. The secondary outcome was metabolite comparison between different sex. We made a 1:1 propensity-score matched comparison between male and female adults by matching participants by age, BMI, serum creatinine level, and hypertension, and then compared their differences in metabolite abundance between male and female adults. All statistical analyses were performed on SAS software (version 9.4; SAS Institute Inc., Cary, NC, USA), and a two-sided *p* value less than 0.05 was defined as statistically significant.

## Results

### Study patient demographics

**Fig. 1:**
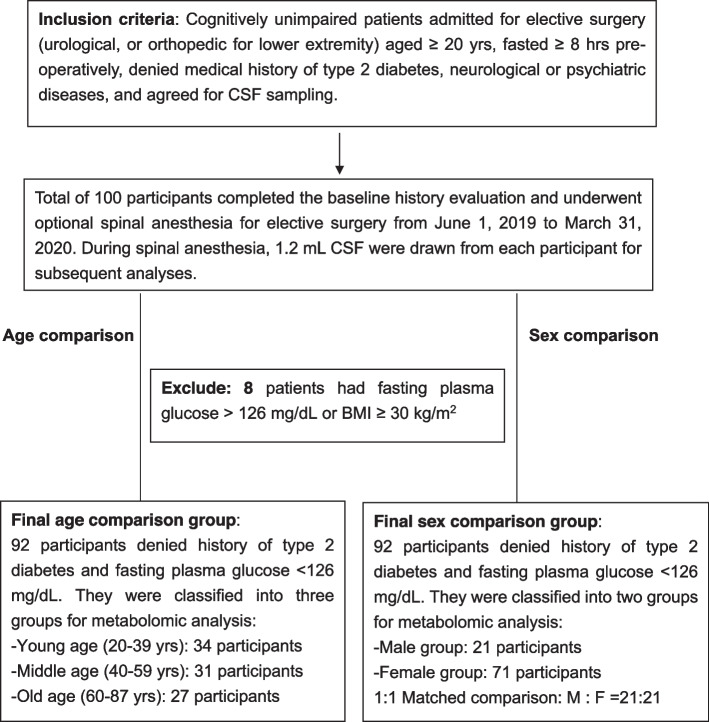
Flow chart for the study design and group separation

Our final cohort included 92 patients, which were divided into the following three age groups: young (*n*=34), middle-aged (*n*=31), and old (*n*=27) groups. The study protocol is shown in Fig. 1. Basic between-group comparisons of the demographic and biochemical parameters are shown in Table 1. Basic demographic comparison of the young, middle-aged, and old age groups showed that patients in the old age group had significantly higher BMI, serum creatinine level, and percentage of medical history of hypertension than those in the other age groups. We adjusted for these significant variables in subsequent between-group comparisons.

**Table 1:** Demographic comparison among young, middle-aged, and old patients

**Group in CSF**	**Young (20–39 y/o) (*n* = 34)**	**Middle (40–59 y/o) (*n* = 31)**	**Old (60–87 y/o) (*n* = 27)**	***p* value**^#^
Male sex, N(%)	4 (11.76%)	7 (22.58%)	10 (37.04%)	0.065
Age (mean ± SD, years)	29.35 ± 6.03	48.39 ± 5.55	68.67 ± 6.08	< 0.001*
BMI (kg/m^2^)	21.05 ± 2.61	22.42 ± 2.78	24.59 ± 2.56	< 0.001*
Fasting blood glucose (mg/dL)	92.11 ± 3.22	98.33 ± 5.86	109.67 ± 20.30	0.525
Serum creatinine (mg/dL)	0.61 ± 0.16	0.64 ± 0.16	0.74 ± 0.26	0.028*
Hypertension	0 (0%)	1 (3.23%)	11 (40.74%)	< 0.001*
Hyperlipidemia	0 (0%)	2 (6.45%)	0 (0%)	0.195
Obesity	0 (0%)	0 (0%)	0 (0%)	NA

*NA* Non-applicable

^#^*p* value was calculated using Chi-square test for categorical variables and the analysis of variance (ANOVA) for continuous variables

^*^p < 0.05

### OPLS-DA score plots of LC–MS signal integrations

**Fig. 2:**
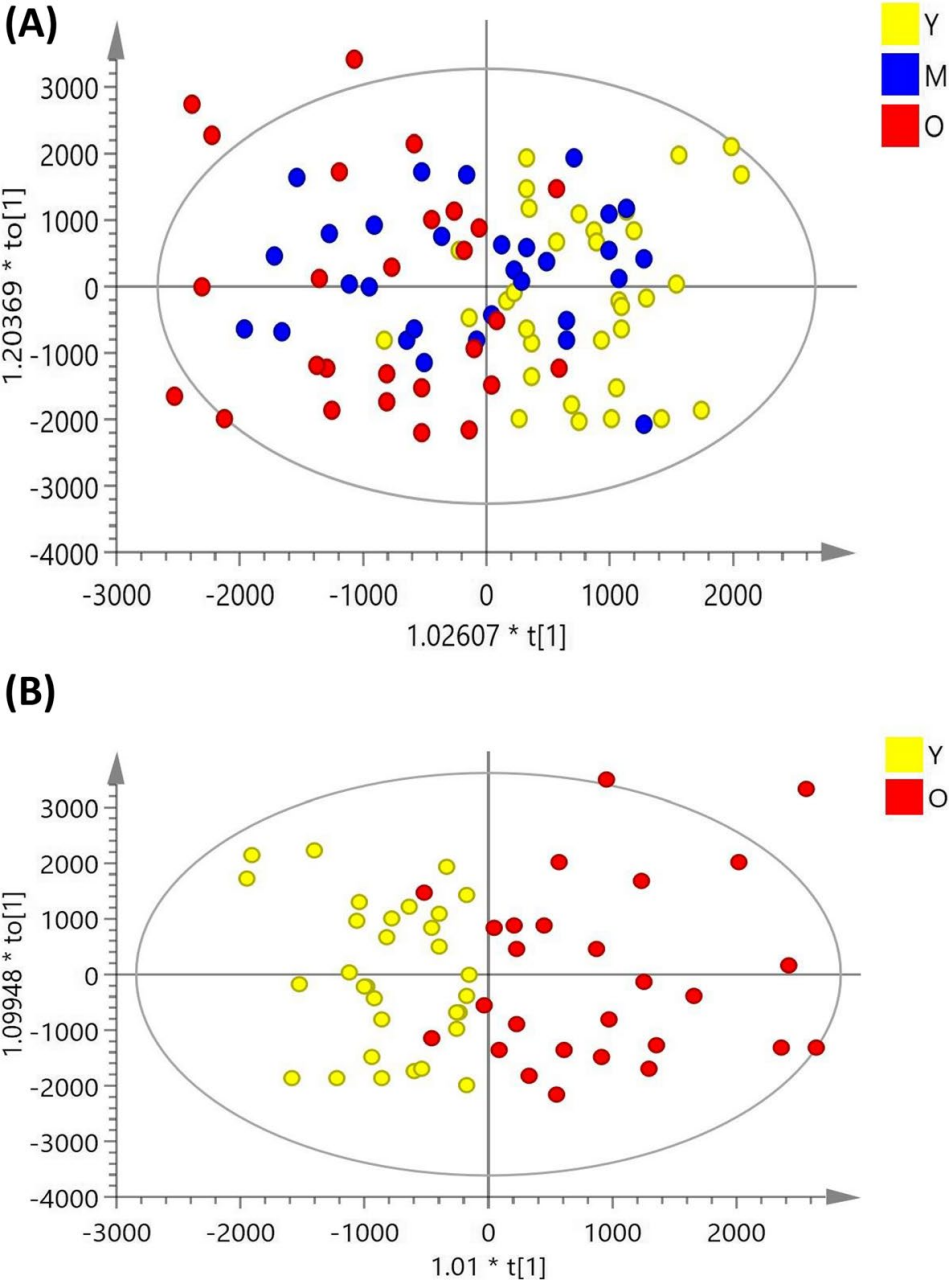
Orthogonal partial least-squares discriminant analysis (OPLS-DA) score plots in CSF samples obtained from (**A**) comparison between young, middle, and old patients (reliability: R^2^X = 0.732, R^2^Y = 0.320, Q^2^ = 0.206). (**B**) young patients versus old patients (reliability: R^2^X = 0.835, R^2^Y = 0.736, Q^2^ = 0.515). The OPLS-DA plots show a clear separation between the young and old groups in CSF samples

The OPLS-DA score plots of the LC–MS signals between age groups are shown in Fig. 2. The OPLS-DA score plots (Fig. 2B) showed a clear discrimination between the old and young groups in the CSF samples (reliability: R^2^X = 0835, R^2^Y = 0.736, Q^2^ = 0.515).

### Comparison of old and young patients

Comparison of the LC–MS signal integration of CSF samples between the young and old groups is shown in Table 2, and comparisons between the other groups are listed in Supplementary Table 1. The LC–MS signal integration for the old age group showed significantly higher levels of pantothenic acid, 5-hydroxyindoleacetic acid (5-HIAA), sucrose, glutamate, and 2-hydroxyglutarate (2-HG) compared to the levels in the young group (adjusted fold change > 1.2, *p* < 0.05). The old age group had lower levels of asparagine and glycerophosphocholine than the young group. These age-related metabolite alterations were visualized in a boxplot for metabolite comparison between young, middle-aged, and old age groups according to LC–MS signal integrations (Supplementary Fig. 1). The metabolite heatmaps of these metabolite differences in the young, middle-aged, and old age groups are shown in Supplementary Fig. 2.

**Table 2:** Comparison of CSF metabolites in young versus old patients

**Metabolites in CSF**	**LC–MS signal integration (mean ± SD) (× 10**^**3**^** a.u.)**		**Adjusted fold change**	**Adjusted *p* value**^a#^
	**Old**	**Young**	**Old/Young**	**Old vs. Young**
**Increased metabolite levels in old patients compared to young patients**				
Pantothenic acid	2287.78 ± 235.62	1300.62 ± 283.32	1.759	0.003*
5-Hydroxyindoleacetic acid	137.82 ± 10.14	81.14 ± 12.19	1.698	< 0.001*
Sucrose	48.22 ± 3.98	36.43 ± 4.79	1.323	0.032*
Glutamate	15.53 ± 0.94	12.42 ± 1.13	1.250	0.017*
2-hydroxyglutarate	97.29 ± 6.26	78.84 ± 7.53	1.234	0.033*
Pseudouridine	355.90 ± 11.66	306.73 ± 14.02	1.160	0.002*
Cysteine	35.29 ± 1.40	30.97 ± 1.69	1.140	0.025*
**Decreased metabolite levels in old patients compared to young patients**				
Isoleucine	4.25 ± 0.53	4.56 ± 0.64	0.932	0.667
Asparagine	149.02 ± 7.57	184.07 ± 9.11	0.809	0.001*
Glycerophosphocholine	63.04 ± 4.91	83.57 ± 5.90	0.754	0.002*

^a^*p* value was adjusted for sex, body mass index (BMI), hypertension, and serum creatinine. ^#^ *p* value was calculated using two sample *t* test. *p < 0.05

### Correlation between metabolite abundance and aging

**Fig. 3:**
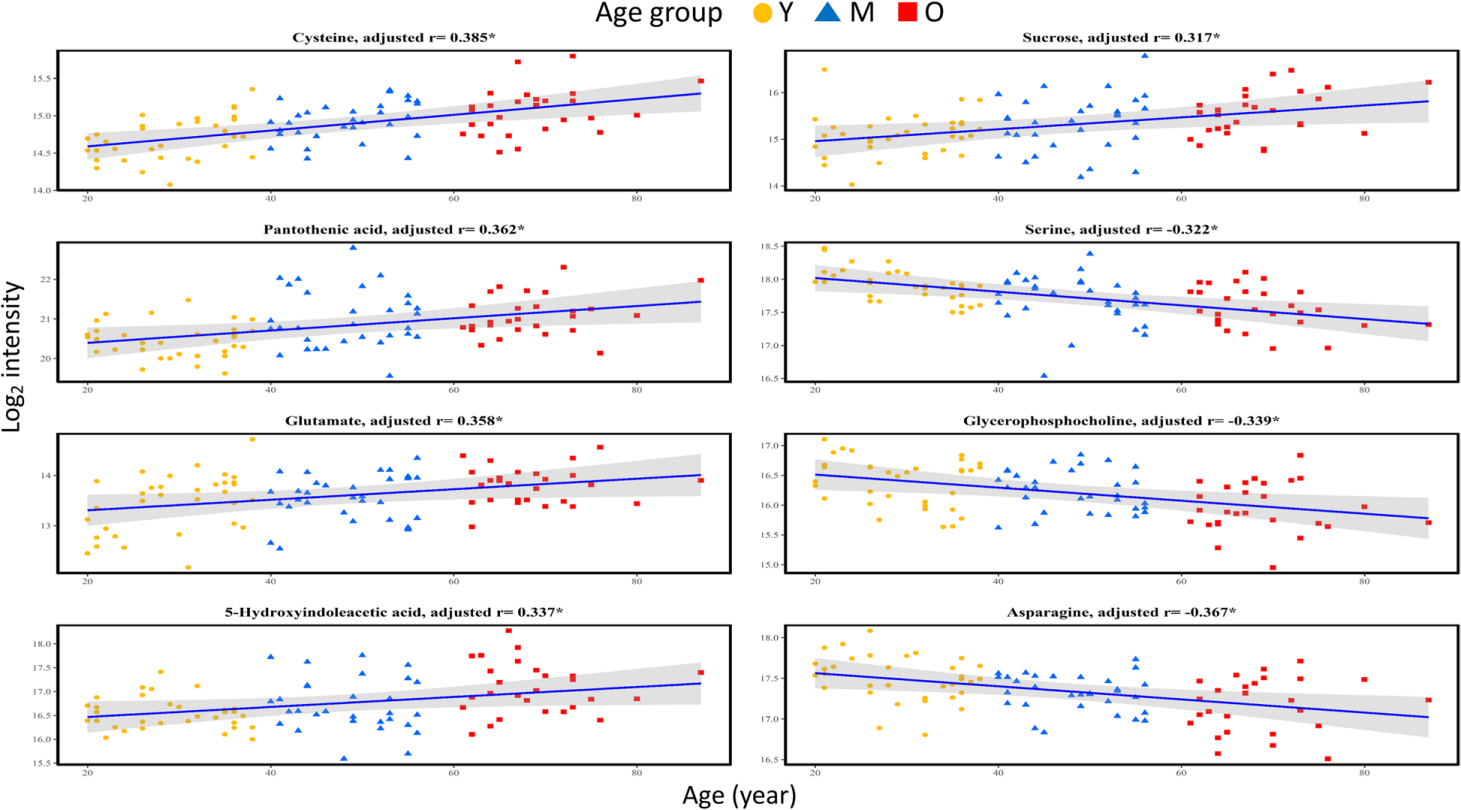
Scatter plot of log_2_ transformed metabolite abundance and its correlation with age in metabolites with significant correlation with aging. Value refers to log_2_ transformed intensity and age is given in years. The colored zones refer to 95% confidence intervals of the regressions

The correlations of the log_2_ transformed metabolite abundance with age are listed in Table 3. Cysteine, pantothenic acid, 5-HIAA, glutamate, aspartic acid, pseudouridine, sucrose, and 2-HG were positively correlated with age (adjusted *r* > 0,* p* < 0.05), whereas α-ketoglutarate (α-KG), glutamine, serine, glycerophosphocholine, and asparagine were negatively correlated with age (adjusted *r* < 0, *p* < 0.05). Figure 3 shows scatter plots of the log_2_ transformed metabolite abundance and their specific correlations within the three age groups. To identify aging-correlated biomarkers in the cerebral circulation, we constructed metabolite combinations using CSF metabolites with significant discrimination between young and old patients. We then compared the AIC and AUC values and adjusted ORs of these combinations for discriminating between the young and old age groups utilizing stepwise ANOVA and multivariate analysis, and the results are shown in Table 4. The combination of asparagine, cysteine, glycerophosphocholine, pantothenic acid, sucrose, and 5-HIAA had comparatively lower AIC values, significant adjusted ORs, and the highest AUC (0.982) for discriminating between the young and old age groups.

**Table 3:** Correlation of log_2_ transformed CSF metabolite abundance with age within different age group

Correlation of log_2_ transformed metabolite abundance with age	All	Young	Middle	Old
**Significantly changed metabolites in CSF**	**Adjusted r**^**a#**^	**Adjusted r**^**a#**^	**Adjusted r**^**a#**^	**Adjusted r**^**a#**^
Cysteine	0.385*	0.426*	0.333	0.123
Pantothenic acid	0.362*	-0.116	0.045	0.413*
Glutamate	0.358*	0.462*	0.282	-0.028
5-Hydroxyindoleacetic acid	0.337*	-0.077	-0.051	-0.077
Aspartic acid	0.331*	0.584*	0.469*	-0.086
Pseudouridine	0.322*	0.107	0.314	-0.042
Sucrose	0.317*	0.097	0.201	0.377
2-hydroxyglutarate	0.289*	0.137	0.032	-0.161
Mannitol	0.284*	0.778*	0.196	-0.015
Cystathionine	0.218*	-0.031	0.311	0.079
Taurine	0.082	0.159	0.145	-0.035
Isoleucine	-0.021	0.125	0.069	-0.035
G1P	-0.039	0.349	0.037	-0.442*
Lactate	-0.173	-0.212	-0.302	0.100
Alpha-ketoglutarate	-0.221*	-0.279	-0.578*	-0.211
Glutamine	-0.255*	-0.300	-0.225	-0.053
Serine	-0.322*	-0.655*	-0.095	-0.225
Glycerophosphocholine	-0.339*	-0.242	-0.224	-0.028
Asparagine	-0.367*	-0.224	-0.221	0.147

^a^Adjusted for sex, body mass index (BMI), hypertension, and serum creatinine

^#^r was calculated using regression model

^*^p < 0.05

**Table 4:** Association of altered CSF metabolite combinations for discriminating between the young and old age groups

Comparison	Young vs. Old			
**Significantly changed metabolites in CSF**	**AIC**	**AUC**	**Adjusted OR**^**a#**^	**Adjusted* p* value**^**a**^
Pantothenic acid	64.688	0.845	1.000	< 0.001*
Cysteine	67.213	0.817	1.000	0.002*
5-Hydroxyindoleacetic acid	69.629	0.786	1.000	0.002*
Pseudouridine	71.602	0.806	1.000	0.002*
Asparagine	71.692	0.776	0.999	0.002*
Glycerophosphocholine	72.392	0.783	0.999	< 0.001*
Sucrose	76.168	0.760	1.000	0.008*
Asparagine, Pantothenic acid	48.552	0.931	2.718	< 0.001%
Asparagine, Pantothenic acid, 5-Hydroxyindoleacetic acid	39.861	0.959	2.718	< 0.001*
Asparagine, Cysteine, 5-Hydroxyindoleacetic acid, Pantothenic acid	34.956	0.975	2.718	< 0.001*
Asparagine, Cysteine, Pantothenic acid, 5-Hydroxyindoleacetic acid, Sucrose	34.597	0.979	2.718	< 0.001*
Asparagine, Cysteine, Pantothenic acid, 5-Hydroxyindoleacetic acid, Sucrose, Glycerophosphocholine	34.655	0.982	2.718	0.001*

*AIC* Akaike information criterion, *OR* Odds ratio

^a^Adjusted for sex, body mass index (BMI), hypertension, and serum creatinine

^#^Odds ratio was calculated using logistic regression model

^*^p < 0.05

### Correlation of metabolite abundance with aging between the sexes

**Fig. 4:**
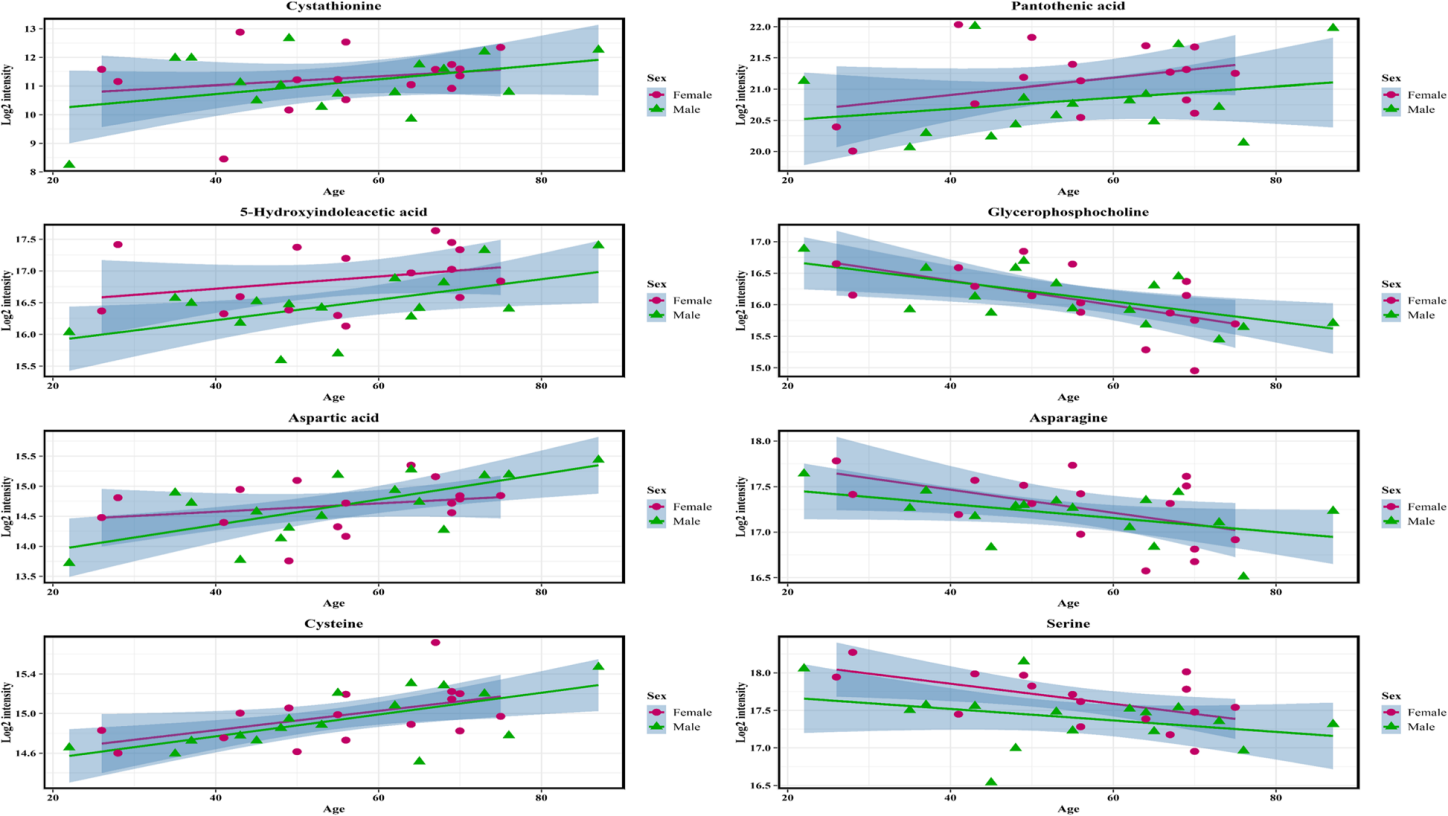
Scatter plot of log_2_ transformed metabolite abundance and its correlation with age within male and female group. Value refers to log_2_ transformed intensity and age is given in years. The colored zones refer to 95% confidence intervals of the regressions

The aging process might differ between males and females; therefore, we divided our cohort according to sex, and the demographic comparison is shown in Table 5. We then calculated the correlation between metabolite abundance and aging for the two sexes (Table 6). More metabolites were significantly correlated with aging in women than in men, and some metabolites had significantly positive correlations with aging in both sexes, including 5-HIAA, aspartic acid, cysteine, and pseudouridine. Scatter plots of the log_2_ transformed metabolite abundance and their correlations with aging within the male and female groups is shown in Fig. 4.

**Table 5:** Demographic comparison between different sex

Group in CSF	Original				1:1 propensity-matched comparison^a^		
	**Male (*n* = 21)**	**Female (*n* = 71)**	*p* value^#^		**Male (*n* = 21)**	**Female (*n* = 21)**	*p* value^#^
Age (mean ± SD, years)	55.90 ± 18.13	44.76 ± 15.95	0.007*		55.13 ± 17.01	55.50 ± 15.13	0.948
BMI (kg/m^2^)	23.88 ± 2.39	22.16 ± 3.05	0.019*		23.02 ± 1.85	24.10 ± 3.68	0.309
Fasting blood glucose (mg/dL)	101.13 ± 16.51	118.00 ± 14.14	0.225		103.23 ± 14.8	118.00 ± 14.14	0.276
Serum creatinine (mg/dL)	0.85 ± 0.24	0.60 ± 0.14	< 0.001*		0.78 ± 0.14	0.77 ± 0.16	0.872
Hypertension	7(33.33%)	5(7.04%)	0.005*		4(25%)	5(31.25%)	1.000
Hyperlipidemia	1(4.76%)	1(1.41%)	0.406		1(6.25%)	1(6.25%)	1.000
Obesity	0 (0.00%)	0 (0.00%)	NA		0 (0.00%)	0 (0.00%)	NA

*NA* Non-applicable, *BMI* Body mas index

^#^*p* value was calculated using Chi-square test for categorical variables and the analysis of variance (ANOVA) for continuous variables

^a^matched with age, BMI, serum creatine and hypertension

^*^p < 0.05

**Table 6:** Comparison of altered CSF metabolites within different sex and their correlation with aging process

**Metabolites in CSF**	**Male**		**Female**	
	**Log2 transformed LC–MS signal integration (mean ± SD) (a.u.)**^**a**^	**Correlation of log2 transformed abundance with age (r**^**a#**^**)**	**Log2 transformed LC–MS signal integration (mean ± SD) (a.u.)**^**a**^	**Correlation of log2 transformed abundance with age (r**^**a#**^**)**
5-Hydroxyindoleacetic acid	16.44 ± 0.14	0.570*	16.86 ± 0.09	0.328*
Cystathionine	11.14 ± 0.37	0.559*	11.51 ± 0.24	0.183
Aspartic acid	14.73 ± 0.15	0.551*	14.73 ± 0.09	0.279*
Cysteine	14.96 ± 0.08	0.547*	14.93 ± 0.05	0.343*
Pseudouridine	18.25 ± 0.06	0.517*	18.33 ± 0.04	0.277*
Glutamate	23.09 ± 0.04	0.448	23.11 ± 0.03	0.343*
2-hydroxyglutarate	16.36 ± 0.11	0.459	16.44 ± 0.07	0.262*
Pantothenic acid	20.59 ± 0.17	0.258	20.80 ± 0.11	0.384*
Sucrose	15.26 ± 0.15	0.398	15.26 ± 0.09	0.245*
Mannitol	18.68 ± 0.09	0.316	18.66 ± 0.06	0.319*
Uric acid	18.59 ± 0.14	0.132	18.39 ± 0.09	0.161
Taurine	12.71 ± 0.24	-0.153	13.32 ± 0.15	0.159
Serine	17.45 ± 0.08	-0.376	17.76 ± 0.05	-0.261*
Glycerophosphocholine	16.15 ± 0.11	-0.368	16.08 ± 0.07	-0.314*
Asparagine	17.31 ± 0.08	-0.413	17.34 ± 0.05	-0.358*

^a^Adjusted for body mass index (BMI), hypertension, and serum creatinine

^#^r was calculated using regression model

^*^p < 0.05

### Metabolite differences between males and females

**Fig. 5:**
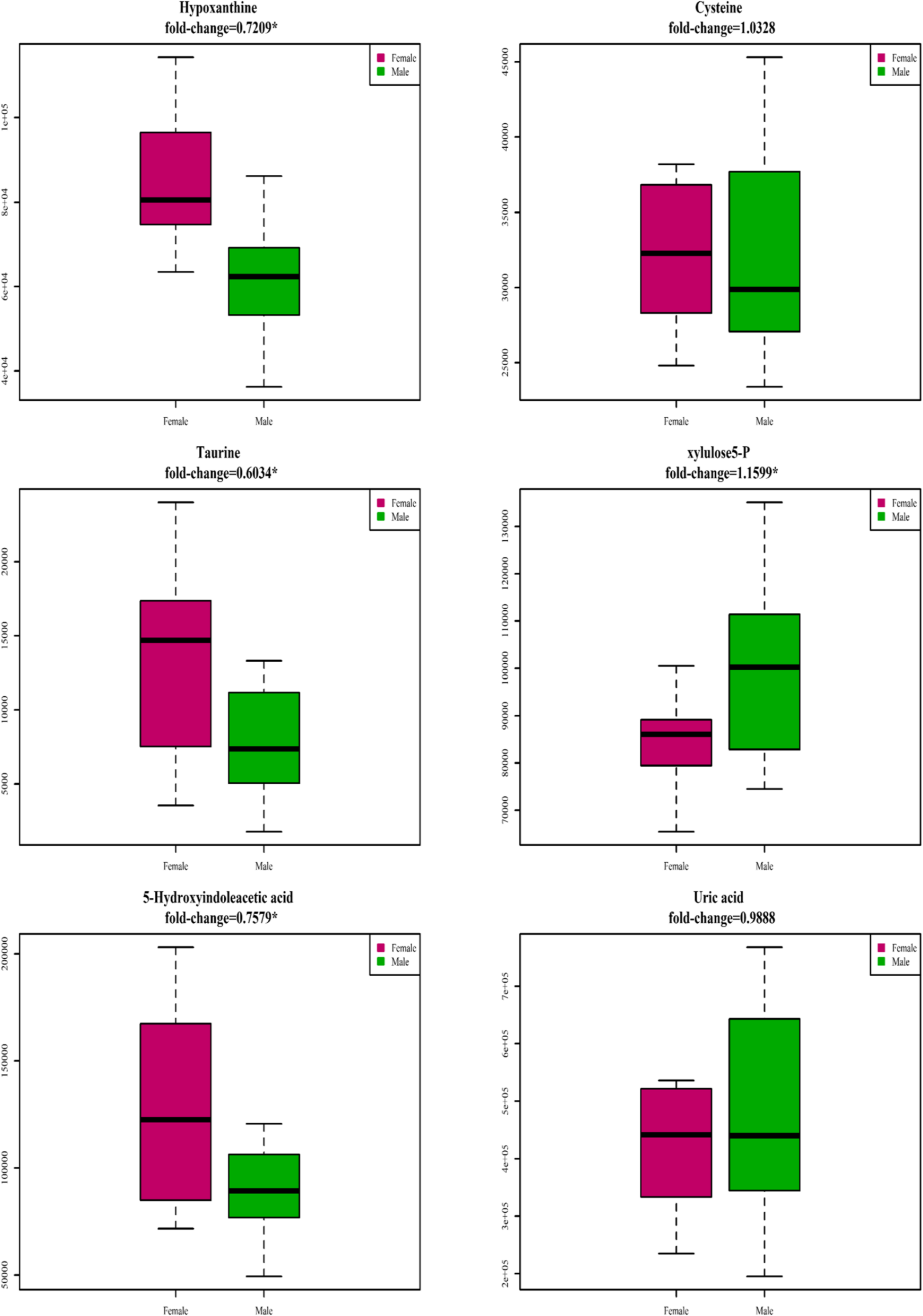
Boxplot for 1:1 propensity-matched metabolite abundance comparison between male and female group. Propensity-score matched by age, body mass index (BMI), hypertension, and serum creatinine

To further compare the differences in CSF metabolites between males and females, we utilized propensity score-matched comparison by 1:1 matching according to age, BMI, serum creatinine, and hypertension to eliminate these confounders. After propensity score matching, no obvious between-group differences were observed (Table 5). Between-sex comparisons of CSF metabolites are shown in Table 7. Female adults had significantly higher levels of hypoxanthine, 5-HIAA, and taurine than the matched males (fold change of male/female < 0.8, *p* < 0.05), and the boxplots of the propensity-matched between-sex comparisons are shown in Fig. 5.

**Table 7:** Comparison of CSF metabolites between male and female patients using 1:1 propensity-matched comparison by matching with age, body mass index (BMI), hypertension, and serum creatinine

**Metabolites in CSF**	**LC–MS signal integration (mean ± SD) (× 10**^**3**^** a.u.)**		**fold change**	***p* value **^**#**^
	**Male**	**Female**	**Male/Female**	**male vs. female**
**Increased metabolite levels in male patients compared to female patients**				
Xylulose 5-P	99.91 ± 19.54	86.14 ± 12.08	1.159	0.022*
**Decreased metabolite levels in male patients compared to female patients**				
Uric acid	470.37 ± 181.03	475.69 ± 235.73	0.988	0.943
cysteine	31.94 ± 6.52	32.98 ± 7.06	0.968	0.661
uridine	5252.33 ± 784.14	5958.56 ± 1208.74	0.881	0.059
serine	179.38 ± 48.81	211.11 ± 50.56	0.849	0.081
5-hydroxyindoleacetic acid	95.72 ± 34.07	126.29 ± 42.50	0.758	0.032*
Hypoxanthine	61.45 ± 14.11	85.24 ± 15.41	0.721	0.001*
Taurine	7.95 ± 3.58	13.18 ± 5.91	0.603	0.005*

^#^*p* value was calculated using two sample *t* test

^*^p < 0.05

### Altered metabolic pathways in the cerebral circulation during aging

**Fig. 6:**
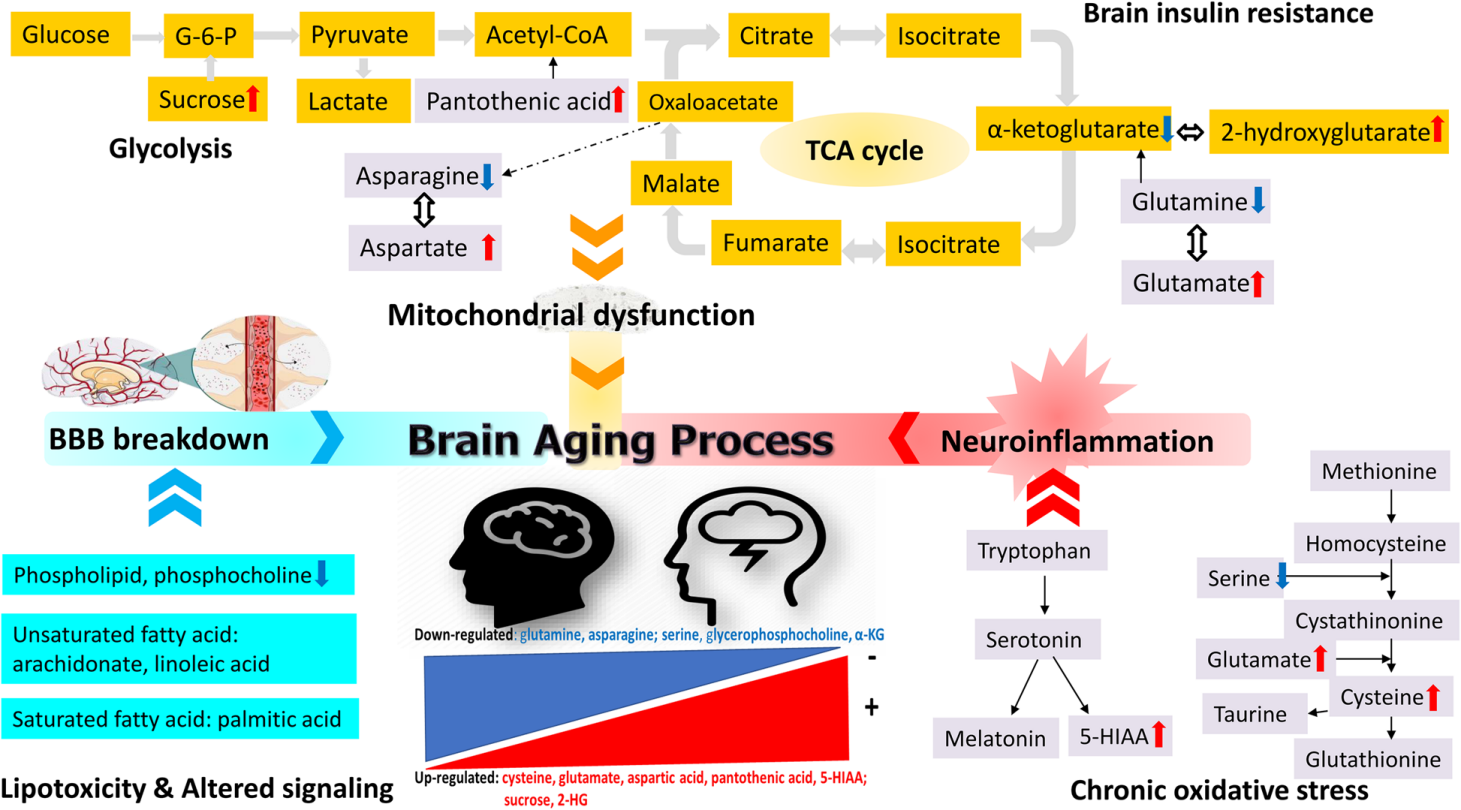
A schematic diagram illustrates these profiled CSF metabolites change during brain aging process. These aging-related CSF metabolomic change might suggest possible blood-brain barrier(BBB) breakdown, neuroinflammation, and mitochodrial dysfunction in aged brain circulation. Metabolites marked in blue refer to those which decreased in the elderly, while metabolites marked in red refer to those which increased with increasing age

The metabolites that are altered during aging are mainly related to amino acids, lipids, neurotransmitters, and energy metabolism. Enrichment analysis of these altered metabolites showed significantly higher enrichment ratios for aminoacyl-tRNA biosynthesis, glutamine and glutamate metabolism, butanoate metabolism, and pantothenate and CoA biosynthesis. Pathway analysis of these age-correlated metabolites showed higher correlations with alanine, aspartate, and glutamate metabolism; arginine biosynthesis; glutamine and glutamate metabolism; and aminoacyl-tRNA biosynthesis. The involved metabolic pathways and their profiled metabolic changes during brain aging process of these age-correlated metabolites are depicted in Fig. 6. The significant aging-related changes in asparagine, glycerophosphocholine, cysteine, pantothenic acid, sucrose, serine, and 5-HIAA levels in fasting CSF samples suggest downregulation of cell membrane, amino acid, and neurotransmitter metabolism and mitochondrial dysfunction in the aged brain.

## Discussion

This prospective cohort study intended to profile the metabolomic signature in CSF during aging in cognitively unimpaired Taiwanese adults using LC–MS. Metabolomic analysis of fasting CSF samples showed significantly higher levels of pantothenic acid, 5-HIAA, sucrose, glutamate, and 2-HG and lower levels of asparagine and glycerophosphocholine in the old age group than in the young group. The combination of asparagine, cysteine, glycerophosphocholine, pantothenic acid, sucrose, and 5-HIAA was strongly correlated with aging, with an AUC of 0.982 to discriminate the old age group from the young group. We also found higher levels of taurine, hypoxanthine, and 5-HIAA in CSF samples from females than in CSF samples from males using a 1:1 propensity-score matched comparison. These aging-related CSF metabolites indicate decreased lipid, amino acid, neurotransmitter, and energy metabolism, suggesting possible BBB breakdown, neuroinflammation, and mitochondrial dysfunction in aged brain circulation.

This LC-MS metabolomic analysis of healthy aging is an extension of our previous NMR analysis of aging metabolomics. The two most common metabolomic analytic methods are NMR spectroscopy and mass spectrometry-based metabolomics. While NMR spectroscopy can identify core metabolites in key metabolic pathways, mass spectrometry-based metabolomics can identify low abundance metabolites with wide detection range and excellent sensitivity [11]. In our earlier publication on CSF metabolomics of aging using NMR platform, we found a combination of CSF metabolites of citrate, lactate, leucine, tyrosine, and valine correlated superiorly with aging, implying higher anaerobic glycolysis and mitochondrial dysfunction in the cerebral circulation during aging [20]. In this later performed LC–MS analysis of aging metabolomics in a largely different patient population (only 31 patients were both included in previous NMR and this LC–MS study), we identified more aging-correlated metabolites and had a different insight into healthy brain aging. In this study, we identified several CSF metabolites that were positively correlated with the aging process (pantothenic acid, 5-HIAA, glutamate, aspartic acid, pseudouridine, sucrose, and 2-HG) and several that were negatively correlated with aging (α-KG, glutamine, serine, glycerophosphocholine, and asparagine). These metabolites are mainly carbohydrates, amino acids, and phospholipids, which are involved in cell membrane turnover, redox reactions, neurotransmitter metabolism, and mitochondrial respiration. The difference of identified aging-correlated metabolites between our previous NMR study and this current LC–MS analysis may be contributed by different metabolomic techniques, group classification, and diverse patient population. Combining the previous NMR and this LC–MS metabolomic results, we can have better comprehension of CSF metabolome and a deeper insight into the delicate regulation of glucose hypometabolism, BBB breakdown, neuroinflammation, and mitochondrial dysfunction during brain aging.

Pantothenic acid, also known as vitamin B5, is the most altered metabolites in this LC–MS analysis. Pantothenic acid is an essential trace nutrient important for the synthesis of coenzyme A, which plays an important role in the tricarboxylic acid (TCA) cycle, fatty acid metabolism, acetylcholine, and myelin synthesis [26]. A recent nested case–control study of serum metabolomics found increased levels of free fatty acids, acylcarnitines, and pantothenic acid in patients with early cognitive decline over a 12-year follow-up [27]. Our finding of increased pantothenic acid in aging brains might be explained by enhanced acetyl-CoA transport to fuel dysfunctional mitochondria or an accumulation due to dietary intake of pantothenic acid in elderly patients [28].

5-HIAA is a major metabolite of serotonin. A previous CSF metabolomics study of subjects with AD and mild cognitive impairment found that these patients had elevated levels of methionine and 5-HIAA [29]. Glutamate is an excitatory neurotransmitter that plays an important role in cyclic adenosine monophosphate signaling to enhance insulin secretion [30]. Elevated oxidative stress in the elderly might reduce glutamine synthesis and impair the glutamate-glutamine cycle in astrocytes in the aging brain, leading to glutamate accumulation and subsequent neurodegeneration and cognitive dysfunction [31].

Cysteine, a non-essential amino acid, is metabolized from the methionine and can be converted to glutathione, an important antioxidant and free radical scavenger [32]. Cysteine inhibits mitochondrial respiration by limiting intracellular iron bioavailability via an oxidant-based mechanism [33]. Increased cysteine levels in the elderly may imply higher cysteine toxicity, a major driver of age-related mitochondrial dysfunction [33]. Aspartic acid racemization reflects age-dependent accumulation of abnormal proteins in various tissues and is correlated with the aging of long-lived proteins; thus, it might play an important role in aging-related diseases [34].

Pseudouridine, also known as the ‘fifth nucleotide’ of RNA, is an isomer of the nucleoside uridine and plays an important role in the metabolism of purine nucleosides, muscle amino acids, and organic acids [9]. RNA pseudouridination is the most common post-transcriptional RNA modification that is dynamically remodeled in response to cellular stress and the regulation of mRNA pseudouridine epitranscriptome is a potential pharmacological target for various human diseases [35]. A human urine metabolomics study of the aging process found a decline in pseudouridine levels in the elderly [9]. However, we found higher pseudouridine levels in CSF samples from older individuals; thus, the aging-related changes in CSF pseudouridine require further clarification.

2-HG is structurally similar to α-KG and is associated with tumorigenesis and neurological dysfunction [36]. 2-HG extended the lifespan of *C. elegans* by binding to and inhibiting ATP synthase, thereby decreasing mitochondrial respiration and mTOR signaling [36]. Mutation of isocitrate dehydrogenase (IDH) in tumor cells resulted in conversion of α-KG to 2-HG; subsequent accumulation of 2-HG led to epigenetic dysregulation via inhibition of α-KG-dependent histone and DNA demethylase [36]. In our study, we observed elevated 2-HG accumulation with aging in human CSF samples, which might be attributed to decreased mitochondrial respiration in the aged brain. α-KG is a key metabolite in the TCA cycle and is involved in various fundamental cellular functions, such as collagen synthesis, epigenetic regulation, and stem cell proliferation. The levels of α-KG change upon fasting, exercise, and aging [37]. A recent study in C57BL/6 mice found that an α-KG-supplemented diet extended the lifespan of middle-aged female mice and increased the healthspan of both sexes [37]. Besides, α-KG extended the lifespan of *C. elegans* by inhibiting ATP synthase and TOR signaling and suppressed chronic inflammation in female mice [37]. Decreased α-KG levels in the CSF of elderly suggest decreased mitochondrial oxidative phosphorylation in the aged brain.

Asparagine, a non-essential amino acid, is essential for the brain development and function, and an asparagine synthetase deficiency can lead to congenital microcephaly and neuronal damage [38]. Asparagine is converted to aspartate through deamination, which is a form of non-enzymatic post-translational modification and protein aging process that has been associated with neurodegenerative diseases [39]. The decreased asparagine levels in the aged human CSF samples in our study might be explained by protein aging.

Glycerophosphocholine, a glycerophospholipid with choline as a headgroup, is the main component of biological membranes and is a reservoir for second messengers. Alteration of the glycerophospholipid composition of neural membranes may change neural membrane permeability and is correlated with neurodegenerative diseases [40]. A large-scale cohort study comparing 150 healthy individuals using non-targeted plasma LC–MS metabolomics revealed the levels of phospholipids (phosphocholine), phosphoserine, and prostaglandin changed with aging [11]. A recent systematic review of 39 studies on memory and gait decline with aging found that the five most age-correlated plasma or serum metabolites were sphingolipids, fatty acids, phosphatidylcholines, amino acids, and biogenic amines [41]. These results suggest that lipid metabolism is closely associated with aging by affecting membrane permeability, energy metabolism, signaling pathways, and gene expression; the decreased glycerophosphocholine in the CSF of the old age group in our study suggests defective cell membrane turnover and BBB breakdown in the aged brain.

The altered levels of asparagine, cysteine, pantothenic acid, sucrose, 5-HIAA, and glycerophosphocholine in the CSF samples of the older group in our study suggest decreased lipid, amino acid, neurotransmitter, and energy metabolism in the aged brain. A previous Swedish study of CSF metabolomics found significant positive associations between age and acetylcarnitine, glutarylcarnitine, hippurate, 5-hydroxytryptophan, isoleucine, ketoleucine, methionine, and pipecolate and negative correlations between age and methylthioadenosine and 3-methy-ladenine [19]. A recent CSF metabolomic analysis of a cohort at Duke Medical Center, consisting of 129 healthy individuals, found 11 metabolites positively correlated with age (4-hydroxyphenyllactic acid, 7-methylxanthine, cysteine, guanosine, glutathione, gamma tocopherol, kynurenine, methionine, tryptophol, uric acid, and xanthine), and one metabolite negatively associated with age (3-O-methyldopa) [32]. Most of these age-correlated CSF metabolites are involved in amino acid metabolism or redox reactions, implying higher oxidative stress and defected oxidative phosphorylation in the aged brain circulation, which is in line with our CSF metabolomics results. Our profile of altered CSF metabolites during aging is somewhat different from those in previous publications. A possible explanation for this might involve differences in patient selection, analytical technologies, and diverse populations.

Examining samples from healthy adults is of paramount importance as it could help us understand the healthy aging process and how aging trajectories differ between the sexes, thus enabling us to identify pathogenic phenomena and targeted therapeutics for neurodegenerative diseases. Exploring the sex differences between male and female adults was our secondary outcome. Previous plasma metabolomics studies exploring sex differences in aging found that the significant differences between men and women could be attributed to five metabolic pathways: primary bile acid biosynthesis, lysine degradation, fatty acid biosynthesis, linoleic acid metabolism, and the pentose phosphate pathway [11]. Another small metabolomics analysis of human CSF from 32 cognitively healthy older volunteers found significantly higher acylcarnitine levels in males and higher taurine levels in females [42]. Taurine, a major constituent of bile, has multiple biological functions, including conjugation of bile acids, antioxidation, osmoregulation, membrane stabilization, and modulation of calcium signaling [42]. Taurine was also found significantly higher in females in several CSF metabolomic analyses, which is in line with our result [42, 43]. A Duke CSF metabolomics study found that men had significantly higher levels of cysteine, uric acid, and N-acetyl-serotonin, while women had significantly higher 5-HIAA levels [32]. The sex difference in 5-HIAA levels could reflect enhanced serotonin transporter function and serotonin metabolism in the female brain [32]. Hypoxanthine, a purine derivative, is an intermediate in nucleic acid metabolism, and in humans, adipose tissue is a major source [44]. Sex differences in cognitive decline and AD susceptibility have been reported; approximately two-thirds of patients with AD are women, and previous studies found a higher risk of AD in female mice [45, 46]. The sex difference in AD susceptibility might be correlated with age-related changes in female brains due to the metabolic effects of pregnancy and menopause, which might signal the hypometabolic phenotype of AD [46]. In our CSF metabolomic profiling of sex difference in aging, we found more metabolites that were significant correlated with age in females, which might be explained by more profound metabolic changes in female brains. The identified sex-specific metabolites in our study, i.e., higher hypoxanthine, taurine, and 5-HIAA levels in women, might be associated with higher levels of adipose tissue release, bile acid, and neurotransmitter metabolism in women.

Our CSF metabolomics analysis of cognitively healthy adults revealed decreased phospholipid, amino acid, neurotransmitter, and energy metabolism during aging, suggesting aging-related metabolic changes in BBB breakdown, neuroinflammation, and mitochondrial dysfunction, which is in line with previous studies on brain aging [47]. With better comprehension of CSF pathophysiology, we could expand our understanding of the brain’s orchestrated regulation and develop novel strategies to improve brain health during aging. Emerging findings suggest that to tackle these metabolic changes during brain aging, brain rejuvenating strategies, such as eliminating conditions with deleterious metabolic effects, including diabetes and obesity, and practicing intermittent bioenergetic challenges, such as intellectual activities, dietary energy restriction, and physical exercise are needed [5]. Cell culture and animal model-based studies have shown that intermittent bioenergetic challenges can reactivate neuroplasticity, bolster mitochondrial respiration, and stimulate mitochondria biogenesis and autophagy [5]. Other brain rejuvenation strategies in model animals, such as heterochronic parabiosis or administration of young plasma seemed to restore brain function in aged mice [47]. A recent animal study found a novel strategy for restoring hippocampal myelination: infusion of young CSF, which restored oligodendrogenesis and rejuvenated the aged mouse brain via activation of oligodendrocyte progenitor cells by fibroblast growth factor 17 [48]. These studies indicate that we could improve brain health by preventing metabolic diseases and introducing intermittent bioenergetic challenges. Someday, we might even be able to restore brain neurogenesis to rescue cognitive decline during brain aging and neurodegeneration.

To the best of our knowledge, this CSF metabolomic study is the first to profile the LC–MS metabolomic signature of healthy brain aging in the Asian population. Compared to previous CSF metabolomics studies, our metabolomic cohort has a homogeneous ethnicity, adequate patient number, and case information [19]. Our CSF sampling methodology during spinal anesthesia enabled us to detect real-time CSF metabolomic alterations without additional patient discomfort or risk. Additionally, since we intended to determine the metabolomics profile during the healthy aging process, we minimized potential bias by excluding patients with cognitive problems, diabetes, and obesity and adjusting for significant confounders during comparison. Therefore, no extreme metabolite outliers were detected in our final LC–MS dataset, which enabled minimization of individual bias.

Although this study was metabolically well characterized, it had some limitations. First, we only collected CSF samples once from the study participants, so we could not compare matched metabolomic information from other samples, such as plasma or urine. In fact, it is not yet known whether the changes in the analyzed metabolites represent neutral changes with age, sudden dynamic stress response, or merely individual variations. [11] Considering aging process as a continuous evolving phenomenon, age-related changes may be better detected by a longitudinal correlational approach, and that longitudinal follow-up recording is our next research project. Second, our enrolled participants had demographical differences in sex, BMI, serum creatinine, and medical diseases between different groups, thus might compromise the analysis even though these confounders have been adjusted before comparison. Besides, due to the complex nature of aging process and many factors that could affect the dynamic CSF metabolome, such as exercise, diet, lifestyle, sleeping, and medications, our results require further validation in larger cohort or further longitudinal-designed study to eliminate these confounders. Third, our results of metabolomic profiling of the aging process in CSF samples might reflect the combined effects of metabolic dysfunction, decreased CSF turnover, and BBB breakdown in the aging brain circulation, but these individual variables could not be quantified due to methodological limitations.

## Conclusions

In this cross-sectional cohort study of LC–MS metabolomic profiling of human CSF samples from cognitively healthy adults in the Taiwanese population, we presented novel insights into metabolic dysregulation in cerebral circulation during aging. These identified CSF metabolite changes in aging process are involved in metabolism of lipid, amino acid, neurotransmitter, and mitochondrial respiration. The profiled aging metabolomic changes might imply defected cellular signalling, BBB breakdown, neuroinflammation, and mitochondrial dysfunction in the aging cerebral circulation. Furthermore, a combined CSF alteration of asparagine, cysteine, glycerophosphocholine, pantothenic acid, sucrose, and 5-HIAA displayed a superior correlation with the aging process, which may provide clues for healthy brain aging and deserve further investigation for causal relationships.

## Supplementary Information

**Additional file 1:** **Supplementary Materials**: **Table S1**. Comparison of CSF metabolite abundance in different age group.

**Additional file 2:** **Figure S1**. Boxplot for metabolite comparison between young, middle, and old age group.

**Additional file 3:** **Figure S2**. Metabolite heatmaps in CSF samples. (A) Young versus old group, (B) Young versus middle versus old group.

## Data Availability

The raw data could be available by connecting the corresponding author.
