# Supplementary material for: Exploring the aging process of cognitively healthy adults by analyzing cerebrospinal fluid metabolomics using liquid chromatography-tandem mass spectrometry
Source: BMC Geriatr. 2023 Apr 5;23:217. doi: 10.1186/s12877-023-03939-6 (PMC10077689; doi:10.1186/s12877-023-03939-6)

**Supplementary Materials**:

**Supplementary Table 1. Comparison of CSF metabolites in different age group**

| **Comparison** | **Old age/ Young age** | | **Middle age /Young age** | | **Old age/Middle age** | |
| --- | --- | --- | --- | --- | --- | --- |
| **Significantly changed metabolites** | **Adjusted fold change ^a^** | **Adjusted p value ^a^**^#^ | **Adjusted fold change ^a^** | **Adjusted *p* value ^a^**^#^ | **Adjusted fold change ^a^** | **Adjusted *p* value ^a^**^#^ |
| Pantothenic acid | 1.759 | 0.003* | 1.709 | <0.001* | 1.029 | 0.832 |
| 5-Hydroxyindoleacetic acid | 1.699 | <0.001* | 1.229 | 0.095 | 1.382 | 0.005* |
| Sucrose | 1.323 | 0.032* | 1.176 | 0.142 | 1.125 | 0.297 |
| Glutamate | 1.250 | 0.017* | 1.055 | 0.507 | 1.185 | 0.049* |
| 2-hydroxyglutarate | 1.234 | 0.032* | 1.282 | 0.002* | 0.963 | 0.644 |
| Pseudouridine | 1.160 | 0.002* | 1.025 | 0.540 | 1.131 | 0.007* |
| Cysteine | 1.139 | 0.025* | 1.120 | 0.016* | 1.017 | 0.741 |
| Isoleucine | 0.932 | 0.667 | 1.039 | 0.756 | 0.896 | 0.475 |
| cystathionine | 1.619 | 0.138 | 1.179 | 0.588 | 1.372 | 0.267 |
| Uric acid | 1.001 | 0.994 | 1.022 | 0.798 | 0.979 | 0.836 |
| Lactate | 0.944 | 0.158 | 1.022 | 0.484 | 0.923 | 0.039* |
| Serine | 0.887 | 0.073 | 0.885 | 0.022* | 1.003 | 0.961 |
| Cysteine glutathione disulfide | 0.848 | 0.221 | 0.778 | 0.026* | 1.090 | 0.548 |
| Asparagine | 0.809 | 0.001* | 0.874 | 0.006* | 0.925 | 0.225 |
| Glycerophosphocholine | 0.754 | 0.002* | 0.874 | 0.051 | 0.863 | 0.118 |

^a^ Adjusted for sex, body mass index (BMI), hypertension, and serum creatinine, ^#^ *p* value was calculated using two sample t test. * *p* < 0.05.


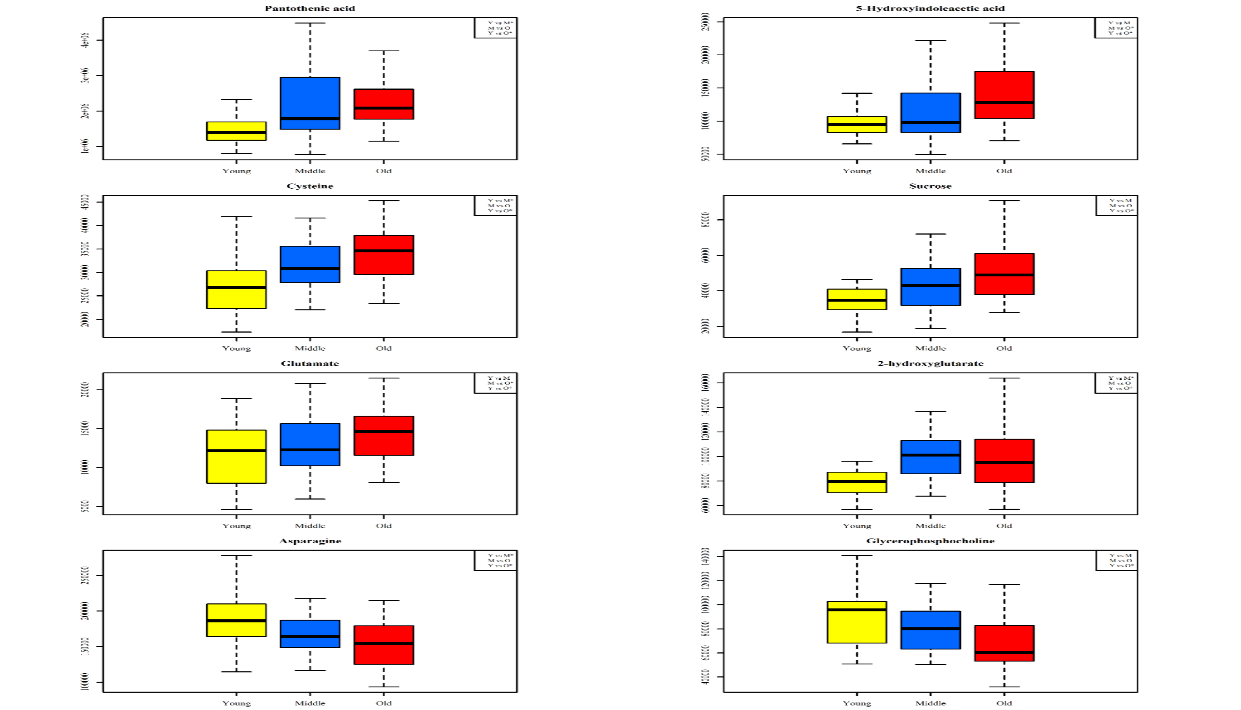
**Figure S1**: Boxplot for metabolite comparison between young, middle, and old age group.

**Figure S2**: Metabolite heatmaps in CSF samples. (A) Young versus old group, (B) Young versus middle versus old group.


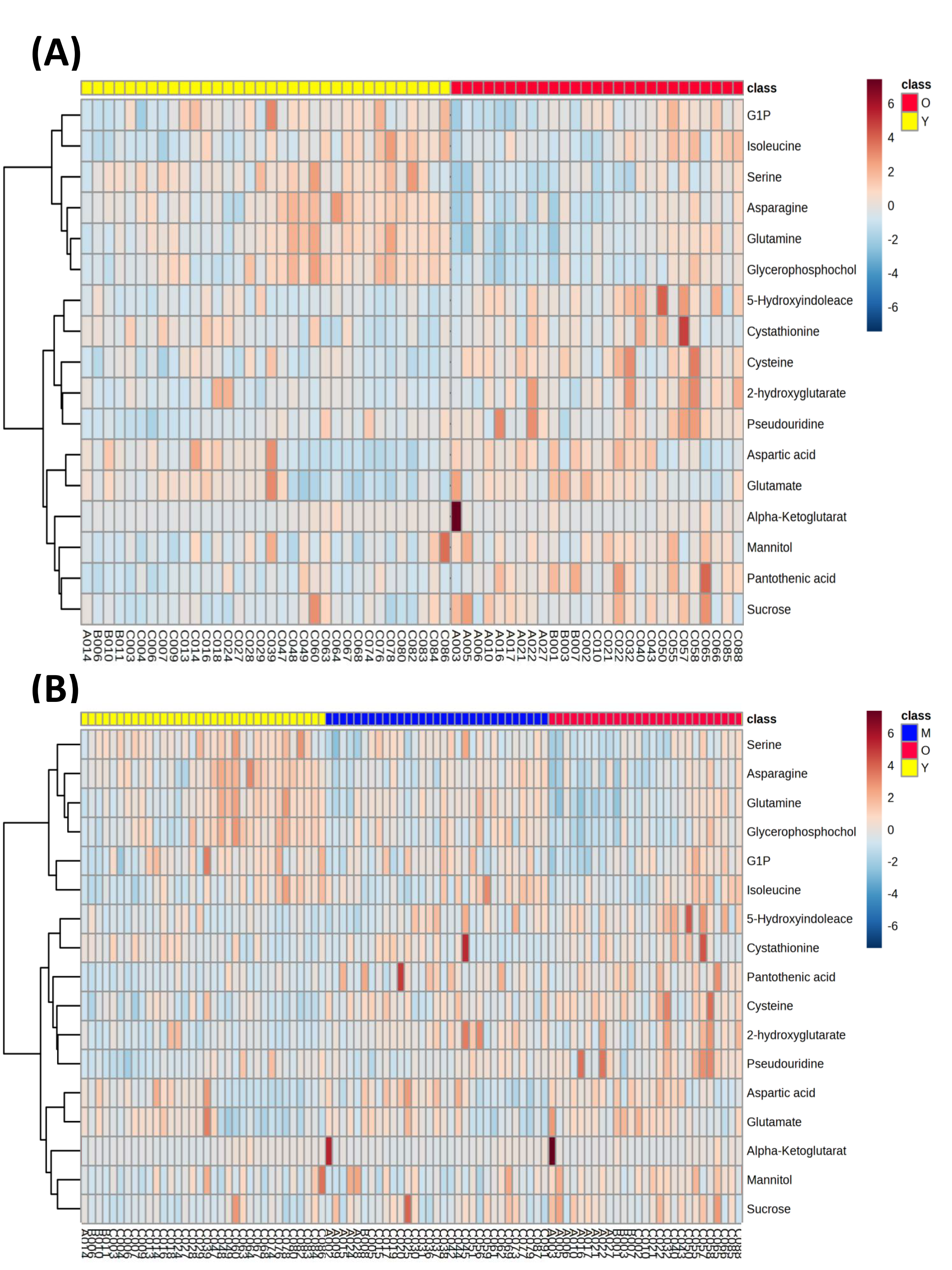

Supplement: Supplementary file 1 — Additional file 1: Supplementary Materials: Table S1. Comparison of CSF metabolite abundance in different age group. [file 12877_2023_3939_MOESM1_ESM.docx]
