# Supplementary figures and images for: Exploring the aging process of cognitively healthy adults by analyzing cerebrospinal fluid metabolomics using liquid chromatography-tandem mass spectrometry
Source: BMC Geriatr. 2023 Apr 5;23:217. doi: 10.1186/s12877-023-03939-6 (PMC10077689; doi:10.1186/s12877-023-03939-6)

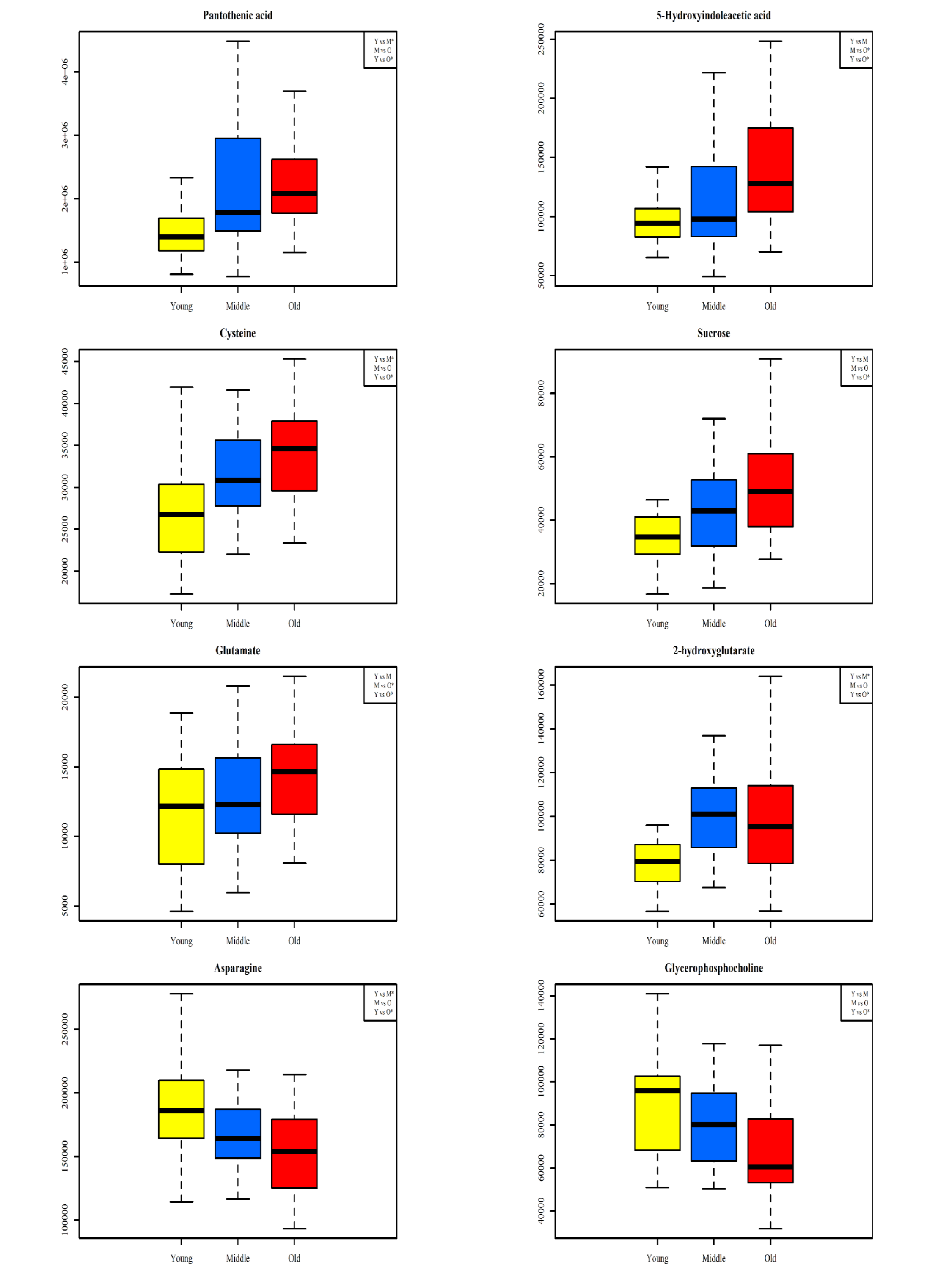

Supplement: Supplementary file 2 — Additional file 2: Figure S1. Boxplot for metabolite comparison between young, middle, and old age group. [file 12877_2023_3939_MOESM2_ESM.tif]

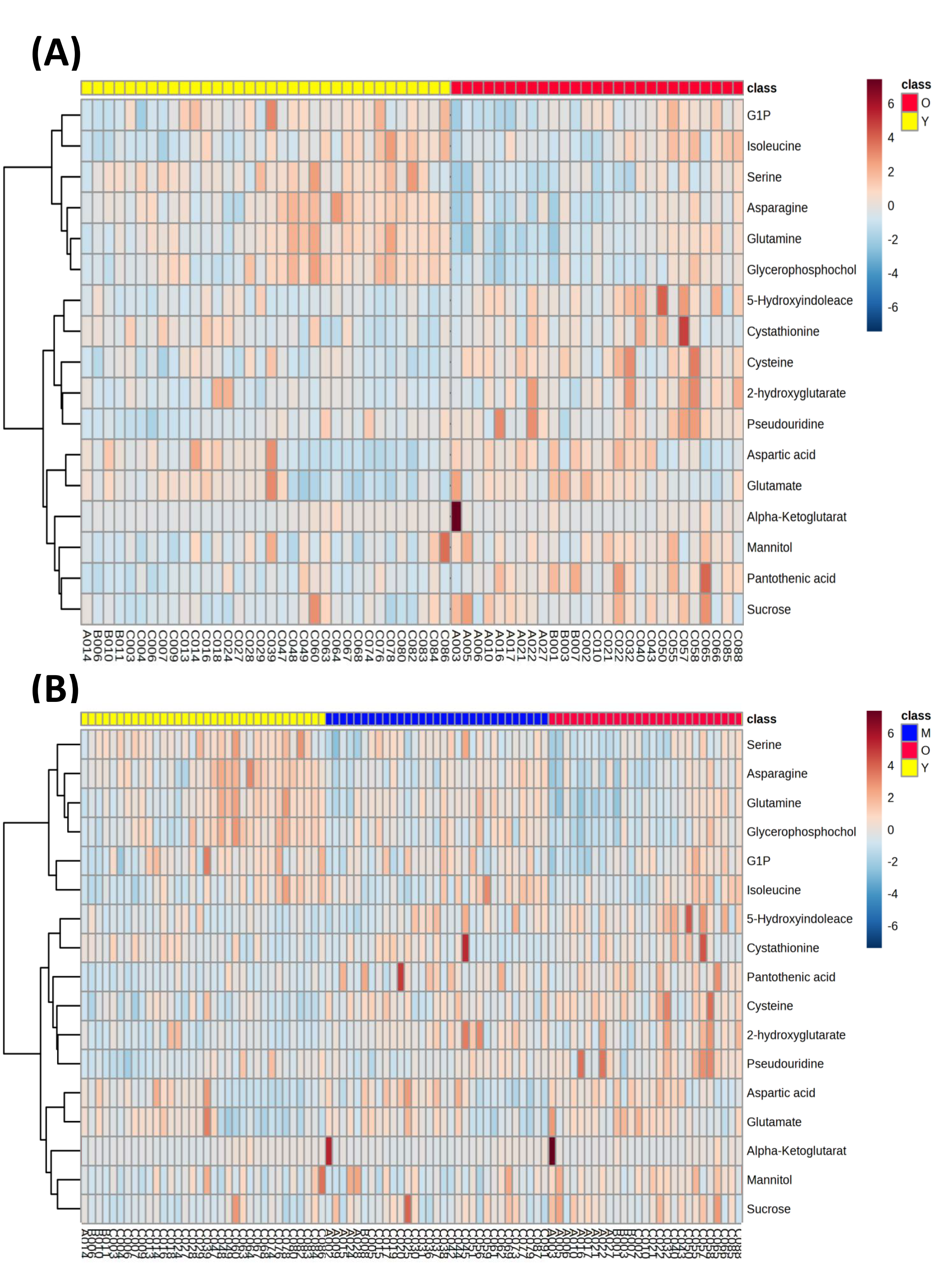

Supplement: Supplementary file 3 — Additional file 3: Figure S2. Metabolite heatmaps in CSF samples. (A) Young versus old group, (B) Young versus middle versus old group. [file 12877_2023_3939_MOESM3_ESM.tif]
